# Severe Tophaceous Polyarticular Gout: A Case Report and Review of Literature

**DOI:** 10.7759/cureus.79045

**Published:** 2025-02-15

**Authors:** Kamyr K Rios Santos, Farah Chohan, Rachell E Simo, Javier B Chambi-Torres, George Michel

**Affiliations:** 1 Internal Medicine, Larkin Community Hospital, South Miami, USA; 2 Psychiatry, Larkin Community Hospital, South Miami, USA

**Keywords:** colchicine, glucocorticoids, gout, gouty arthritis, hyperuricemia, methotrexate, nsaids, pegloticase, placebo, tophaceous gout

## Abstract

Gout is a common form of inflammatory arthritis that affects a significant portion of the population. It is characterized by joint pain, tenderness, and morning stiffness which is more common with acute episodes. Gout is associated with the deposition of monosodium urate crystals, a consequence of hyperuricemia, where serum urate concentrations exceed a critical threshold of 6.8 mg/dL. While many patients are underexcreters of uric acid, some progress to tophaceous gout, marked by severe tissue deposits of monosodium urate (MSU) crystals. This condition typically develops after a decade of untreated symptomatic gout but can occasionally be present at initial diagnosis.

In this case, we present a 43-year-old male with a long-standing history of chronic gouty arthritis, who developed an acute exacerbation involving the right shoulder, elbow, and wrist. The patient’s work as a cruise line mechanic limited his access to regular healthcare resulting in recurrent gout flares over the past few years, complicated by tophi in all four extremities. Despite initial concerns, the serology was negative for rheumatoid arthritis. His management included colchicine, non-steroidal anti-inflammatory drugs (NSAIDs), corticosteroids, and long-term urate-lowering therapy with allopurinol with a detailed plan for outpatient follow-up with a rheumatologist. Through this case, we aim to highlight the consequences of delayed treatment, stressing the need for early intervention and consistent management of chronic gout.

We identified a significant trend, showing a dramatic increase in hospital admissions for gout flares reflecting broader trends in patient demographics and healthcare management. Factors contributing to this rise include an aging population with a higher prevalence of obesity and metabolic syndrome, as well as suboptimal gout management in both outpatient and inpatient settings. The rising rates of hospitalizations for gout highlight significant gaps in current management practices, despite the availability of effective treatment options. This warrants an urgent need for healthcare providers to implement effective strategies that optimize outpatient care and enhance adherence to treatment protocols, ultimately improving patient outcomes and reducing healthcare burdens.

## Introduction

Gout is a common inflammatory arthritis, affecting approximately 3.9% of adults in the United States [1]. It arises from the deposition of monosodium urate (MSU) crystals in joints and soft tissues, driven by chronic hyperuricemia [2]. If inadequately treated, gout can progress to tophaceous gout, a severe form of the disease marked by MSU crystal accumulation, leading to joint deformity, functional impairment, and reduced quality of life [3].

Tophaceous gout, which typically develops after years of recurrent flares, poses significant risks, including infection from ulcerating tophi and chronic joint destruction [4]. While acute flares are managed with colchicine, non-steroidal anti-inflammatory drugs (NSAIDs), and corticosteroids [2,5], long-term control through urate-lowering therapies such as allopurinol or febuxostat is essential to prevent progression [5]. In refractory cases, pegloticase, a uricase enzyme, offers significant efficacy in reducing tophi and improving physical function [6].

We present a case of a 43-year-old male with severe polyarticular tophaceous gout resulting from poorly managed hyperuricemia. This case emphasizes the consequences of delayed treatment and highlights the importance of early and consistent management, contributing to the understanding of chronic gout progression and its complications.

## Case presentation

**Figure 1:**
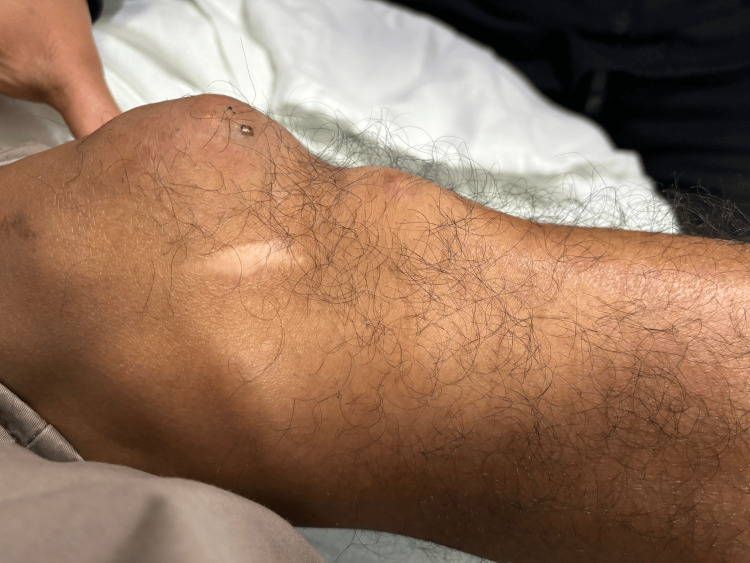
Grossly edematous knee with a large, firm swelling over the knee joint suggestive of a gouty tophus. Erythema and taut skin indicating inflammation, and soft tissue swelling likely due to synovitis in the setting of an acute gout flare were also noted. There is also a small ulcer possibly due to tophi ulceration and spontaneous urate crystal extrusion. There are some knee deformities secondary to chronic gouty arthritis.

**Figure 2:**
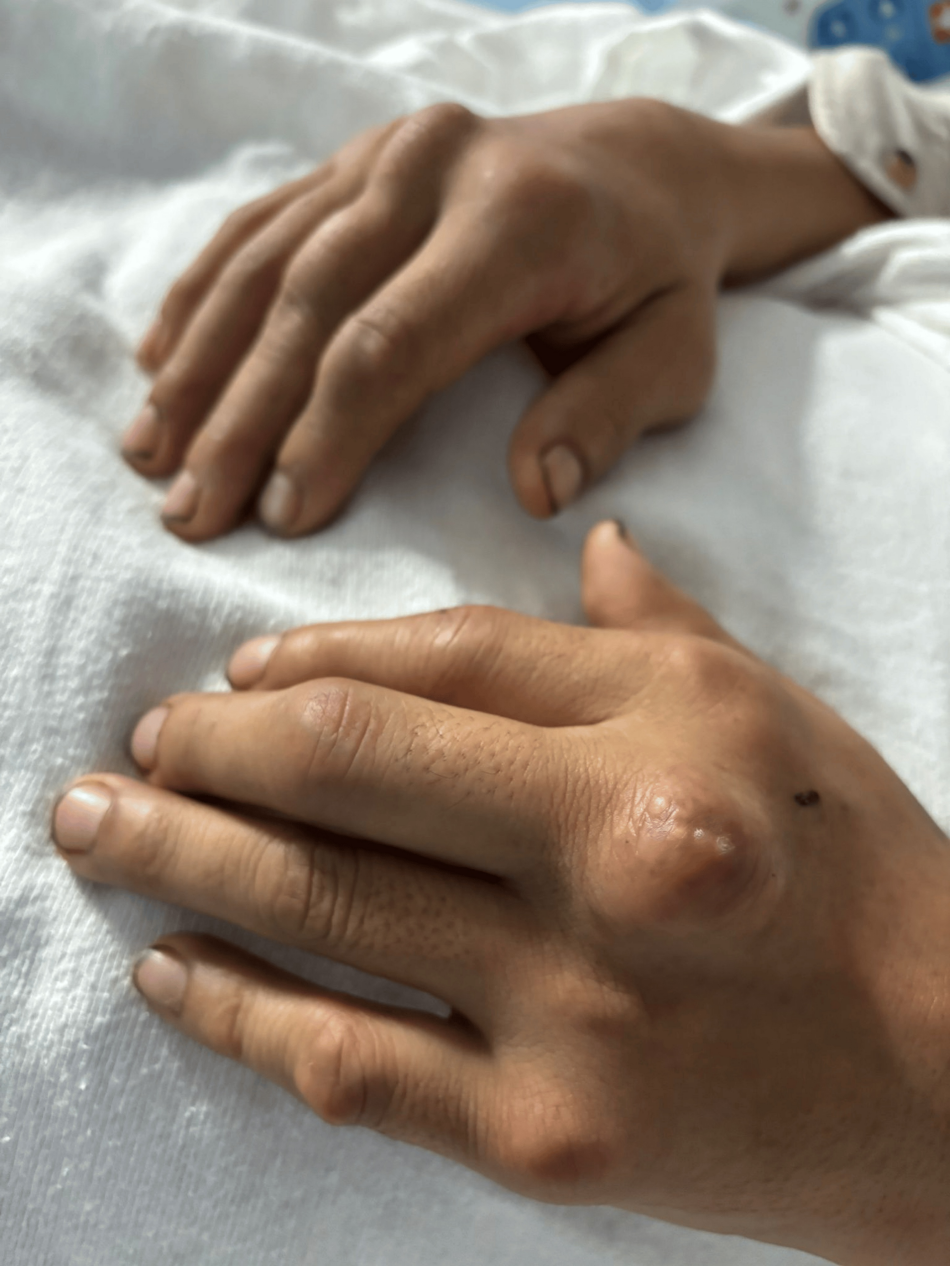
Large, firm, irregularly shaped nodules are visible on the dorsal aspect of the MCP joints. The PCP joints exhibit mild swelling and early signs of nodular changes, suggesting progressive disease. The overlying skin appears tense, shiny, and slightly erythematous, indicating inflammation. One of the tophi exhibits a yellowish-white discoloration which suggests the possible extrusion of urate crystals through the skin. The affected fingers appear swollen and stiff indicating chronic gouty arthritis with joint damage. MCP: metacarpophalangeal PCP: proximal interphalangeal

**Figure 3:**
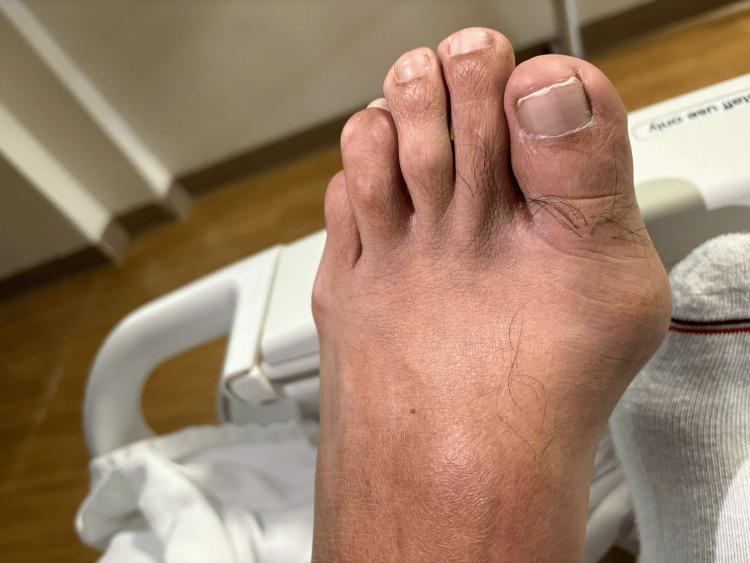
The classic presentation of gout, known as podagra, was observed in the left first metatarsophalangeal joint.

A 43-year-old male with a long-standing history of chronic gouty arthritis (for the past 14 years) presented to the emergency department with acute onset of right shoulder, elbow, and wrist pain that began the previous night. The pain was described as sudden, sharp, and burning, radiating throughout the arm. The right wrist and shoulder exhibited signs of inflammation, including warmth, swelling, and erythema. The patient has a history of gout dating back to 2010 which has been exacerbated by dietary factors and inconsistent hydration. His occupation as a cruise-line mechanic has limited his control over diet and access to healthcare. Over the past two to three years, the patient has received intermittent weeklong courses of medications for gout, but symptoms have recurred after discontinuation of treatment. The patient also reported morning stiffness lasting about an hour and multiple acute flares accompanied by tophi in all four extremities. Examination revealed significant swelling and tenderness in the right shoulder, elbow, wrist, large tophaceous nodules on metacarpophalyngeal joints of bilateral hands (Figure 1), swelling and erythema of right knee (Figure 2) and podagra on left foot (Figure 3). Initial suspicion of underlying rheumatoid arthritis was prompted by the patient's symptoms and radiographic findings. However, rheumatoid arthritis serology was negative. The Rheumatology team was consulted and joint aspiration was performed on the right knee for synovial fluid analysis.

The patient’s hospital course focused on managing symptoms including administering colchicine 0.6 mg orally, Toradol 30 mg intramuscularly for pain control, and intravenous fluids for hydration. Despite these measures, symptoms persisted. Naproxen 500 mg twice a day was added due to persistent pain. Lab results were significant for erythrocyte sedimentation rate (ESR) of 60 mm/hr (elevated), C-reactive protein (CRP) of 8.2 mg/dL (elevated), and uric acid 8.9 mg/dL (elevated) while antinuclear antibodies (ANA), rheumatoid factor, and cyclic citrullinated peptide (CCP) antibodies were negative. Due to persistent pain, two doses of Solu-Medrol 60 mg intravenous push (IVP) were administered over the next couple of days. Once his acute pain resolved, the patient was discharged with allopurinol 100 mg daily, colchicine 0.6 mg daily, Medrol pack for a seven-day taper, and naproxen 500 mg twice daily as needed (PRN) for the next two weeks to manage any breakthrough pain. Omeprazole 20 mg orally before breakfast was prescribed for gastric protection while the patient takes naproxen. Follow-up appointments were scheduled with both the primary care provider and rheumatologist within 10-14 days to reassess the patient's condition and ensure appropriate ongoing management of his gout and any underlying issues.

## Discussion

Inflammatory arthritis encompasses a group of disorders characterized by joint pain, tenderness, swelling, warmth, and morning stiffness lasting more than 30 minutes. Among these, gout is the most common cause affecting approximately 3.9% of the U.S. population. Gout arises from tissue deposition of MSU crystals which occurs due to hyperuricemia. Hyperuricemia is typically defined as a serum urate concentration >6.8 mg/dL [7]. It results from urate overproduction, underexcretion, or a combination of both, with the majority of patients being underexcreters of uric acid. A 24-hour urine collection can help differentiate between overproduction and underexcretion with levels exceeding 800 mg per 24 hours indicating overproduction, and lower values suggesting underexcretion. However, this test is not routinely necessary in clinical practice. It is important to note that all patients experience hyperuricemia at some stage of the disease. However, serum uric acid levels can be normal or low during an acute attack, and individuals with asymptomatic hyperuricemia may never develop clinical symptoms related to urate crystal deposition [8].

A subset of patients with gout may progress to develop tophaceous gout as our patient exhibited. It is a severe form marked by the deposition of MSU crystals in subcutaneous, synovial, or subchondral tissues. Tophaceous gout typically develops after 10 or more years of inadequately treated symptomatic gout, though in rare cases, tophi may be apparent at the time of initial diagnosis. These tophi can ulcerate through the skin discharging a chalky white substance made of concentrated MSU crystals [7]. Besides being disfiguring, tophi can cause functional impairment and lead to bone erosion [9].

If untreated, gout can advance to chronic gouty arthritis, characterized by joint pain even during intercritical periods. In this stage, patients may exhibit symmetric large and small joint involvement resembling rheumatoid arthritis, termed the "pseudorheumatoid arthritis pattern." Redmond et al. in their case report discuss the consequences of poorly managed gout highlighting that longstanding and untreated recurrent attacks of acute gout may lead to advanced tophaceous gout. Monosodium urate deposition can cause severe bony destruction and require digital amputation in rare cases. Severe tophaceous polyarticular gout can occur in the setting of a normal serum urate and inflammatory markers, and if doubt exists, a joint aspiration should be examined by polarized microscopy [10].

Management of gouty arthritis should consider potential drug interactions and comorbidities. Colchicine remains a first-line therapy for both acute gout flares and chronic gouty arthropathy. NSAIDs and glucocorticoids are alternative treatments, especially in cases of severe or polyarticular pain. Ragab et al. stated that colchicine appears much less efficient when given long after the flare onset [11]. Therefore, colchicine is most effective when used less than 24 hours after symptom onset. Given similar efficacy and a lower risk of adverse effects, the American College of Rheumatology (ACR) strongly recommends low-dose colchicine over high-dose colchicine when it is the chosen agent for the management of a gout flare [12]. 

Furthermore, studies done in the past have demonstrated that patients can partially respond to colchicine with improvement in pain but with persistence of swelling. In one of the studies, indomethacin provided relief to a great extent in 48 hours [8]. Other studies have reported the use of long-term colchicine and allopurinol both twice a day along with physical therapy [10]. However, the benefit of the latter in combination with oral therapy could not be reassessed in this study as the patient’s participation in rehabilitation was limited by medical comorbidities. McKenzie et al. studied the use of colchicine for acute gout. Their research focused on the evidence of the benefits and harms of colchicine for the treatment of gout. They found that both high and low-dose colchicine improve pain when compared to placebo. However, they recommended further trials comparing colchicine to placebo or other treatments as no trials have reported the effect of colchicine in populations with comorbidities nor have compared colchicine with other commonly used treatments [13]. 

Urate-lowering therapies (ULTs) form the cornerstone of long-term gout management. These include xanthine oxidase inhibitors such as allopurinol and febuxostat, uricosuric agents like probenecid, and biologics such as pegloticase. Recent studies [14,15] have demonstrated the benefit of combining pegloticase with methotrexate in patients with uncontrolled gout. The MIRROR randomized controlled trial compared pegloticase administered with oral methotrexate versus pegloticase monotherapy. The study showed that 53.8% of patients receiving combination therapy experienced complete resolution of one or more tophi compared to 31% of those treated with pegloticase alone. These findings highlight the importance of maximizing the benefit of therapies in patients with gout, chronic gouty arthritis, and tophaceous gout. A combination of lifestyle modifications and pharmacologic therapy proves to be the most beneficial for long-term management of gout. Lifestyle modifications include purine restricted diet which includes avoidance of high-purine foods including alcoholic beverages, red meat, organ meat, seafood, and some vegetables like spinach and mushrooms and consuming beans, lentils, legumes, low-fat or fat-free dairy, whole grains and adequate hydration.

The ACR and the European League Against Rheumatism (EULAR) advocate for a treat-to-target approach aiming to reduce serum urate levels to below 6 mg/dL or under 5 mg/dL in patients with tophi. The ACR also recommends initiating urate-lowering therapy in patients with gout who present with one or more subcutaneous tophi, radiographic evidence of gout-related damage, or two or more gout flares per year. Allopurinol is the first-line urate-lowering therapy. Importantly, when starting urate-lowering therapy, the mobilization of MSU crystals from tissues may precipitate gout flares [9]. Thus, the therapy can be initiated during an acute flare, provided it is accompanied by anti-inflammatory treatment. If a patient is already on urate-lowering therapy, it should not be discontinued during an acute flare.

## Conclusions

Even though nowadays there is a wide array of medications to treat gout, hospital admissions for gout flares have increased dramatically in recent years. The prevalence of gout has increased in recent years on the background of an aging population with a higher prevalence of obesity and metabolic syndrome, the management of gout is frequently suboptimal in primary care, rheumatology clinics, and inpatient settings; and only a minority of patients achieve the serum urate level required to prevent flares. Modern modalities like dual-energy CT (DECT) can be employed for early diagnosis, however it is currently not available in smaller settings. In light of the above, there is an urgent need to develop strategies to prevent hospitalizations from gout and optimize treatment approaches in the outpatient setting including optimization of pharmacological therapy, combined with lifestyle modifications including a purine-restricted diet. 
